# Mastication and Articulation of Artificial Dentures

**Published:** 1861-11

**Authors:** 


Mastication and Articulation of Artificial Dentures.—*Read before the American Dental Convention, at its annual meeting in New Haven, Ct., August*, 1861, *by* Dr. Burras.—To distinctly understand the subject of mastication,and clearly comprehend the aim and object for which artificial dentures are constructed, it will be necessary to have some knowledge of the parts, particularly of the composition of the tempero-maxillary joint, that is, of the ligaments by which its motions are restrained, and the muscles by which those motions are effected or produced.

The inferior maxillary bone is articulated by its condyles on each side with the glenoid cavity of the temporal bone. This glenoid cavity is bounded anteriorly and superiorly by a prominence called the articulating eminence, and is of some considerable consequence and influence in controlling the motions of the jaw in mastication. From the relative position in which parts composing these joints are disposed, and the intimate connection of the intea-articular cartilage and the condyles, this articulation is very peculiar, somewhat nearly resembling that of the elbow-joint in its motions. There are three ligaments appertaining to this joint. The external lateral, by its superior extremity has a broad attachment to the articular eminence and outer ridge of the glenoid cavity. It extends obliquely backward, and is inserted upon the outer side of the cervix of the lower jaw. The internal lateral is attached by its superior extremity to the inner edge of the glenoid cavity, passing in an oblique direction downward and forward, and is inserted at its inferior extremity to that little bony projection, on the lower jaw, surrounding the posterior maxillary foramen, where the dental vessels and nerves are protected by it from the motions of the internal pterygoid muscle. The stylo-maxillary or suspensory ligament arises from, and is attached to the styloid process of the temporal bone, and is inserted at the angle of the lower jaw. Its use is to restrain the forward motions of the lower jaw, upon the articular eminence of the temporal bone.

The motions of the lower jaw are controlled by the muscles, and are somewhat free and extended. The simple ordinary motions are depression, elevation, protension, and retraction, and I may add, lateral or partial rotation. When the lower jaw is depressed, the condyles are advanced upon the articular eminences, and the angles of the jaw are carried backward; consequently the center of motion is below the condyles, allowing the jaw to be depressed to a greater extent than if the motion of the condyles was restricted to the glenoid cavity.

The only muscle that acts as a depressor of the jaw is the diagastricus. The origin, insertion, direction, and pulley-like attachments are beautifully adapted for the performance of this office. It arises from the root of the mastoid process and os hyoides, and is inserted into the lower and anterior portions of the chin. Its use is to draw the jaw downward, and assist in deglutition.

The elevation of the jaw is performed by four pairs of muscles, the combined power of which is more than most persons would suppose possible, having seen a statement of force exercised by them of 450 lbs. pressure. They are the temporales, masseters, pterygoid, internal and external.

The temporales has its origin from the os frontis, portion of the os temporis, back part of the os malæ, and the temporal portion of the os sphenoides. It is inserted into the coronoid process of the lower jaw. Its use is to move the lower jaw upward.

The masseters have their origin from the malar process of the os maxillare, the lower edge of the os malæ, and the zygomatic process of the os temporales. They are inserted into the base of the coronoid and the condyloid processes. They serve to raise and move the jaw backward and forward.

The pterygoid internus arises from the inner surface of the outer wing of the pterygoid process of the sphenoid bone, and from the process of the os palatæ, that helps to form the pterygoid fossa. It is inserted into the inner side of the lower jaw, near its angle. Its object is to raise the jaw and rotate it to one side. The pterygoid externus arises from the external alæ of the pterygoid process, part of the adjacent maxillary process, and ridge of the temporal process of the os sphenoides. It is inserted at the fore part of the condyloid process, and likewise into the capsular ligament of the lower jaw. Its use is to move the jaw forward and to the opposite side, that is, when the muscle acts singly. When both act in conjunction, the jaw is brought horizontally forward. Its insertion prevents the ligaments of the jaw from being interposed in the peculiarity of its motions.

The process of mastication is performed by the operation of those muscles, the uses of which have just been described, and consists of two distinct actions: biting, or the separation of particles by means of the incisor teeth ; and chewing, or the process of grinding by the molars. By a repeated continuance of this action, the food is ground, comminuted and reduced to a pulp, and is thereby prepared for the act of deglutition or swallowing.

In observing this process, and examining the motion of the lower jaw of children before the teeth are disrobed, there will be considerable deficiency observed in the articulating process of mastication, in not having that free and extended motion which is permitted in the adult. The glenoid cavity being circumscribed to very little more than the size of the condyles, and the articular eminence not being yet formed, consequently the motion of the jaw of infants is confined to simple elevation and depression, with scarcely the least approach to rotation, the condyle being the center of motion in this instance. A sequence almost parallel with this is produced in aged persons who have lost all their teeth, although the state of the mouth, and the circumstances producing this peculiarity are widely different. After the complete removal of all the teeth, and the absorption of the alveolar process has progressed to any great extent, the face will become considerably shortened, in some cases by nearly if not quite the length of the upper and lower teeth, that is, when the mouth is closed to the occlusion of the lower to the upper jaw. When the lower jaw is retained in the position it occupied before the teeth were extracted, the opening between the jaws is sufficient for the introduction of food and most other purposes, and it is seldom necessary to open sufficiently wide to bring the condyles forward on the articular eminences. The operation of mastication in an aged person of this description is very similar to that of an infant, and confined chiefly to the depression and elevation, and not having that alternate lateral motion. When the lower jaw of a person in this condition is closed, the chin describes a large circle and is thrown very much forward, projecting far beyond the upper jaw, so that what little mastication is performed is effected by the sides of the jaws, the only parts that can be brought in contact. In procuring a correct articulation for artificial teeth, the profession has been very much perplexed, and many artificial dentures, otherwise comparatively perfect, have been rendered useless by this operation having been negligently or imperfectly performed. And as there have been, heretofore, no established usage or particular directions adopted by the profession in obtaining correct articulation, I desire to give a mode of practice I have pursued for some years with great satisfaction to myself, and the various members of the profession to whom I have communicated it have given it their *unqualified* approbation. This, in connection with an ardent desire to render as perfect as possible all the manipulations in the practice of our profession, is the only apology I can offer for trespassing on the valuable time of this Convention.

After having procured a perfect adaptation of plate or base to the mouth, set around on it, on the alveolar ridge, a rim of wax, as near the position to be occupied by the artificial teeth as judgment may dictate, being careful to have this rim of wax adhere to the plate or base so as not to be displaced by the operations of taking the articulations; then, while the plate or base is in its correct position in the mouth, direct the patient te swallow, or perform the act of deglutition, and retain the jaw in the same precise position of occlusion as it was on completing this act of swallowing; then, by parting the lips you will perceive the relative position of the jaws, in an exact situation for a correct articulation. As nature herself, by this operation of swallowing, produces an equalization of motion in all the muscles that move the jaw; as there is as much distinctive individuality in the peculiarity of the voice, as there sometimes is in the singularity of the position of the teeth, an imperfect articulation of an artificial denture, producing an effect on the tempero-maxillare joint, will almost entirely obliterate this distinctive personality, and renders a correct articulation of artificial teeth positively necessary to preserve that individuality so peculiar to many persons therefore the more perfect and natural an arrangement of teeth and articulation is procured and preserved, the more complete and harmonious will be that distinctive individuality, which is frequently changed, if not entirely destroyed, by imperfect articulations from artificial dentures.—*Dental Cosmos.*

